# Evaluation of the pharmacokinetic-pharmacodynamic integration of marbofloxacin in combination with methyl gallate against *Salmonella* Typhimurium in rats

**DOI:** 10.1371/journal.pone.0234211

**Published:** 2020-06-04

**Authors:** Biruk Tesfaye Birhanu, Eon-Bee Lee, Seung-Chun Park

**Affiliations:** Laboratory of Veterinary Pharmacokinetics and Pharmacodynamics, College of Veterinary Medicine, Kyungpook National University, Bukgu, Daegu, South Korea; Panjab University, INDIA

## Abstract

Fluoroquinolone resistance in *Salmonella* Typhimurium is becoming a major concern. Hence, an intervention to limit the growth in resistance is inevitable. One way to combat this challenge is through combination therapy. The combination of antibiotics with phytochemicals has become an ideal means of preventing antimicrobial resistance. Recently, in an *in vitro* study, the combination of methyl gallate (MG) with marbofloxacin (MAR) has shown to prevent *Salmonella* Typhimurium invasion. It is also worth to study the effects of plant extracts on the pharmacokinetics of antibiotics. Hence, the objective of this study was to determine the effect of MG on the pharmacokinetics of MAR and pharmacokinetics/pharmacodynamics integration of MG and MAR. The micro-broth dilution method was used to obtain the minimum inhibitory concentration (MIC), and fractional inhibitory concentration (FIC) of MAR and MG. Whereas, the pharmacokinetic was conducted in rats by administering either MAR alone or combined with MG through oral and/or intravenous routes. The results indicated that the MIC of MAR and MG against standard strain *Salmonella* Typhimurium (ATCC 14028) was 0.031 and 500 μg/mL, respectively. The FIC_index_ of the combination of MAR and MG was 0.5. For orally administered drugs, the C_max_ and AUC_24h_ of MAR were 1.04 and 0.78 μg/mL and 5.98 and 6.11 h.μg/mL when MAR was given alone and in combination with MG, respectively. The intravenous administration of MAR showed a half-life of 3.8 and 3.9 h; a clearance rate of 1.1 and 0.73 L/h/kg and a volume of distribution of 5.98 and 4.13 L/kg for MAR alone and in combination with MG, respectively. The AUC_24_/MIC for MAR alone and in combination with MG was 192.8 and 381.9 h, respectively. In conclusion, MG has shown to increase the antimicrobial activity of MAR *in vitro* and *ex vivo* experiments without affecting the pharmacokinetics of MAR in rats.

## Introduction

The broad-spectrum efficacy of fluoroquinolones against a wide range of pathogenic bacterial species has led to its extensive use globally with strict safety measures. However, even with these guidelines, which aim to preserve the efficacy of these drugs, resistance to fluoroquinolones is still emerging at an alarming rate in several bacterial species [1]. Similarly, multiple reports have indicated the development of fluoroquinolone resistance in *Salmonella* along with various antibiotics [2]. Thus, an alternative to these drugs or other means of interventions, including drug combination, are required to limit the growth in resistance against fluoroquinolones.

Drug combinations either with other groups of antibiotics or pairing with nonantibiotic compounds are one approach to overcome drug-resistance [3]. However, a combination might affect the pharmacokinetics (PK) and pharmacodynamic (PD) of an antimicrobial agent [4,5]. Hence, their effectiveness relies on the association between the PK and PD properties of the drugs [6]. Mechanism-based PK/PD are instrumental in keeping the balance between combination therapy and maximizing drug efficacy [7]. The estimate of the clinical efficacy and potency of the drugs depends on the integration of the PK and PD surrogate markers [8]. Hence, the PK/PD predictor indices are the bases for the selection of an appropriate drug and optimize dosage formulation [9,10]. The ratio of the area under the plasma concentration-time curve (AUC) to the minimum inhibitory concentration (MIC) and the ratio of the maximum concentration of the drug in plasma (*C*_max_) to the MIC are the main PK/PD predicting indices for concentration-dependent antimicrobials [11]. However, the AUC/MIC ratio is much preferable to determine the efficacy and potency of marbofloxacin (MAR), which is a widely used antibiotic in veterinary medicine [12].

**Fig 1:**
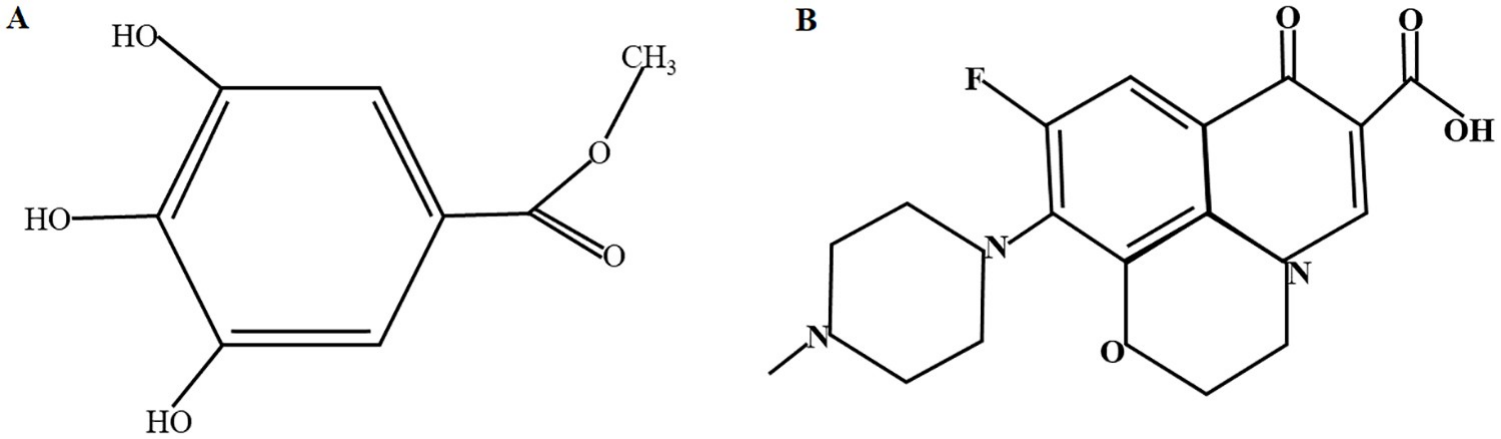
Structural diagram of Methyl gallate (A) and Marbofloxacin (B).

Recently, the utilization of herbal medicine in the treatment of various bacterial infections has been increased due to their efficacy and potency [13]. Hence, the combination of antibiotics with phytochemicals has become an ideal means of preventing antimicrobial resistance. Previous reports indicated that methyl gallate (MG) (Fig 1A), a polyphenol plant, has shown anti-quorum sensing, antioxidant, anti-inflammatory and anti-cancer activity [14,15]. The antimicrobial activity of MG has been shown elsewhere in different bacterial agents [16–21]. This antimicrobial activity also extends to drug resistance pathogens like methicillin-resistant *Staphylococcus aureus* (MRSA) [21], multi-drug resistant *Shigella* species [22] and multiple *Salmonella* strains [19].

Anti-quorum sensing is one of the mechanisms of MG’s antibacterial activity [15]. Recently we have reported the anti-invasive activity and antibacterial mechanism of MG alone and in combination with marbofloxacin (MAR) (Fig 1B) against *Salmonella* Typhimurium [23]. In this study, we presented the synergistic effect of the combination of MG and MAR. These results showed that MG affects the antibacterial and anti-invasive activity of MAR. On the other hand, the PK of MAR has been reported in several previous studies. Another study presented the PK of MG in rats [24]. However, no previous study has been available on the effect of MG on the PK of MAR. Therefore, in this study, we did the first stage of clinical application, investigating the effects of MG on the PK of MAR in clinically healthy rats and computed the PK/PD integration of both compounds.

## Materials and methods

### Chemicals, reagents and antibiotics

All chemicals, reagents, and antibiotics used in this study were procured from Sigma-Aldrich (USA) unless otherwise specified.

### Animal ethics

All procedures on animals were conducted in accordance with the internationally accepted principles for laboratory animal use and care and the ethical requirements, guidance, and approval of the Kyungpook National University, Animal Ethics Committee (2017-0159-1). The experimental animals were used only for this study. At the end of the experiment, all the rats were euthanized as per the guidelines of the Koreans Food and Drug Administration (KFDA) using Carbon Dioxide. Carbon Dioxide was filled in a cage that contains two rats at a flow rate of 5.6 L/min until the rats became unconscious. Finally, the death of the rats was confirmed by cervical dislocation and rats were checked for lack of respiration and faded aye color before disposal.

### Bacterial strains and culture method

Three different strains of *Salmonella enterica* serovar Typhimurium including two field isolates from clinically infected pigs (one susceptible and another intermediately resistant to MAR) and one control (ATCC 14028) were used in this study. *Salmonella* Typhimurium isolation and identification were performed as described previously [25]. For the subsequent processes, the bacteria were cultured at 37 °C in Luria Bertani (LB, Difco, BD, USA) broth or agar plate under aerobic conditions.

### Minimum inhibitory concentration and minimum bactericidal concentration

The minimum inhibitory concentration (MIC) of MAR and MG was determined using the micro-broth dilution method according to the guidelines of CLSI [26]. Briefly, a cation-adjusted Muller Hinton II broth (MHBII, Difco) was used to make a twofold dilution of MAR and MG starting from 1 μg/mL and 2000 μg/mL, respectively. Then, *Salmonella* Typhimurium at a final concentration of 10^5^ CFU/mL was added and cultured overnight at 37 °C under aerobic conditions, and the results were read using a microplate reader (Versa max, Molecular devices, USA) at 600 nm. Whereas, the minimum bactericidal concentration (MBC), was determined by transferring 20 μL of the suspension starting from the MIC onto LB agar plate and incubating for 48 h. Each test was conducted at least three times in duplicate.

### Fractional Inhibitory Concentration (FIC)

The Fractional inhibitory concentration (FIC) was used to determine the effect of the combination of MAR with MG using the Checkerboard method. A final concentration of 10^5^ CFU/mL of the bacteria was added to a different fractional concentration of MAR and MG. The dilution was made in a 96-well plate and incubated aerobically for 24 h at 37 °C. Finally, the results were read both visually and on a plate reader at 600 nm. The FIC index was calculated using the following equation as described previously [27]:

$$\sum FICindex=\frac{C\_{MAR}_{combination}}{MIC\_{MAR}_{alone}}+\frac{C\_{MG}_{combination}}{MIC\_{MG}_{alone}}$$

Where, FIC is fractional inhibitory concentration, C is the lowest concentration of each drug in the combination, and MIC is the minimum inhibitory concentration of each drug alone.

### Pharmacokinetics study

The PK study was conducted on five weeks clinically healthy, male Sprague Dawley rats (OrientBio Inc. Korea) with an average weight of 180–215 gm. The rats were acclimated to the lab condition for one week and provided with ad libitum feed and water throughout the experimental period. Ten rats were used with a four-way crossover method in two weeks interval and received an equal single dosage of MAR (5 mg/kg) alone or in combination with MG (20 mg/kg) through the intravenous (IV) or oral routes. Three-hundred microliter of blood was collected at 0, 0.25, 0.5, 0.75, 1, 2, 4, 8, 12 and 24 h after administration using microvette containing EDTA (Sarstedt, Numbrecht, Germany) from the tail vein. The blood was centrifuged at 2000× g for 15 min at 4 °C and the plasma was stored at -70 °C until used for high-performance liquid chromatography (HPLC) analysis. A non-compartmental analysis with a Linear trapezoidal Linear interpolation calculation method fitted for the orally administered drugs. Whereas, a one-compartment model with first-order input analysis fitted the plasma disposition and selected for plasma concentration analysis of an intravenously administered drugs. The data were analyzed using nonlinear least-squares regression. A comparison of the mean values of the PK parameters of MAR alone and in combination with MG was statistically evaluated using an unpaired t-test.

### High-performance liquid chromatography analysis

MAR concentration in the plasma was measured by HPLC using a Hewlett Packard, Agilent 1100 series. The column temperature was set at 30 °C and the wavelength was detected at 293 nm. Fifty-μL of rat plasma was deproteinated by adding an equal amount of acetonitrile. The sample mix was centrifuged at 14,000 rpm for 7 min and 20 μL of the supernatant was injected into the column. The mobile phase consisted of 80% potassium phosphate buffer (20 mmol, pH = 3), 10% acetonitrile and 10% methanol. The flow rate was set at 1 mL/min [28].

The standard and quality control (QC) samples were prepared using stock solutions of MAR. The accuracy, precision and detection limits of the assays were determined by plotting standard curves of a drug-free plasma spiked with the antibiotic using a high concentration of MAR (50 μg/mL) in plasma and stepwise twofold dilutions (S1 Fig). The samples were also used as a QC for intra- and inter-assays. The antibiotic stock solutions were kept at -20 °C until used. Method validation was determined using the standard deviation and the slope of the calibration curve, which was also used to calculate the detection (LOD) and quantitation limit (LOQ) according to the FDA guidelines [29]. The LOD and LOQ were 0.048 and 0.16 μg/mL, respectively. The correlation of determination (R^2^) of the calibration curve from 0.05 to 50 μg/mL was 0.999. The retention time for MAR was 15.6 min. The intra- and inter-assay coefficients of variation were < 10%. The linear regression equation was Y = 90.567*X– 79.779 (S1B Fig).

### Ex vivo antibacterial effect of plasma samples

The *ex vivo* antibacterial activity of plasma was performed using sampled plasma from treated rats at each time-point. Briefly, 25 μL of plasma from each time-point was added to 225 μL of MHBII broth containing *Salmonella* Typhimurium at a final concentration of 10^6^ CFU/mL. The suspension was incubated at 37 °C and samples were taken at 0, 1, 2, 4, 8, 12 and 24 h. The suspension was serially diluted, plated on LB agar and incubated overnight at 37 °C for bacterial counting. The antibacterial activity was determined using the following equation as previously described for orbifloxacin (Gebru et al., 2009).

$$E=E_{0}+\frac{{\text{I}}_{\text{max}}\times {Ce}^{\gamma }}{I{C_{50}}^{\gamma }+{Ce}^{\gamma }}$$

Where, *E* is the antibacterial effect, measured as the change in bacterial counts (log CFU/mL) in the plasma sample after 24 h of incubation compared to the initial bacterial count; *I*_max_ is the log_10_ difference of the bacterial counts of the control between 0 and 24 h; *E*_0_ is the log_10_ difference of the bacterial counts of the test sample containing MAR between 0 and 24 h, when the count is 10 CFU/mL; Ce is the AUC_0-24_/MIC of the plasma; IC_50_ is the AUC_0-24_/MIC of MAR to produce 50% of the maximal inhibitory effect; and *γ* is the Hill coefficient, which indicates the sensitivity between the exposure and response.

### Pharmacokinetics/Pharmacodynamics (PK/PD) integration indices

The PK/PD integration for MAR was performed as described for fluoroquinolones [12]. The AUC_24_/MIC and C_max_/MIC were calculated from the PK and *in vitro* PD studies. The inhibitory concentration (IC_50_) for the plasma samples were calculated to determine the efficacy of the drugs.

### Dosage formulation

The dosage for MAR alone and in combination with MG was calculated using the following formula.

$$Dosage(mg/kg)=\frac{(\frac{AUC}{MIC})\times CL\times MIC}{fu\times F}$$

Where, AUC is area under the concertation curve; MIC is the Minimum inhibitory concertation; CL is the clearance rate, fu is unbound fraction and F is the bioavailability.

### Statistical analysis

The descriptive statistical analysis and IC_50_ were analyzed using Prism 6 (Graphpad, USA). The PK parameters were analyzed with Phoenix WinNonlin version 8 (Pharsight Corp., St. Louis, MO, USA). Un-paired T-test was used to compare the test groups. P-value <0.05 was considered as statistically significant for all statistics used.

## Results

The MIC and MBC of MAR against both *Salmonella* Typhimurium ATTC 14028 and field strain-2 (S-2) were 0.031 and 0.125 μg/mL, respectively. Whereas, for the field strain 15 (S-15) the MIC and MBC were 0.5 and 2 μg/mL, respectively. The MIC and MBC of MG for all the three *Salmonella* Typhimurium strains were 500 and 1000 μg/mL, respectively (Table 1). The FIC index for the combination of MAR with MG in all three strains was 0.5. The inhibitory concentration of MAR and MG in the combination for ATCC14028 and S-2 were 0.008 and 125 μg/mL while it was 0.125 and 125 μg/mL for S-15 strain, respectively.

**Table 1:** Pharmacodynamics of marbofloxacin and methyl gallate against *Salmonella* Typhimurium strains.

	MIC (μg/mL)		IC_50_		MBC (μg/mL)		FIC_index_
	MAR	MG	MAR	MG	MAR	MG	
**ATCC 14028**	0.031	500	0.03	58.75	0.13	1000	0.5
**S-2**	0.031	500	0.03	53.71	0.13	1000	0.5
**S-15**	0.5	500	0.275	25.64	2	1000	0.52

MIC, Minimum inhibitory concentration; MBC, Minimum bactericidal concentration; IC50, inhibitory concentration of 50% of the population; FIC_index_, Fractional Inhibitory concentration index

**Fig 2:**
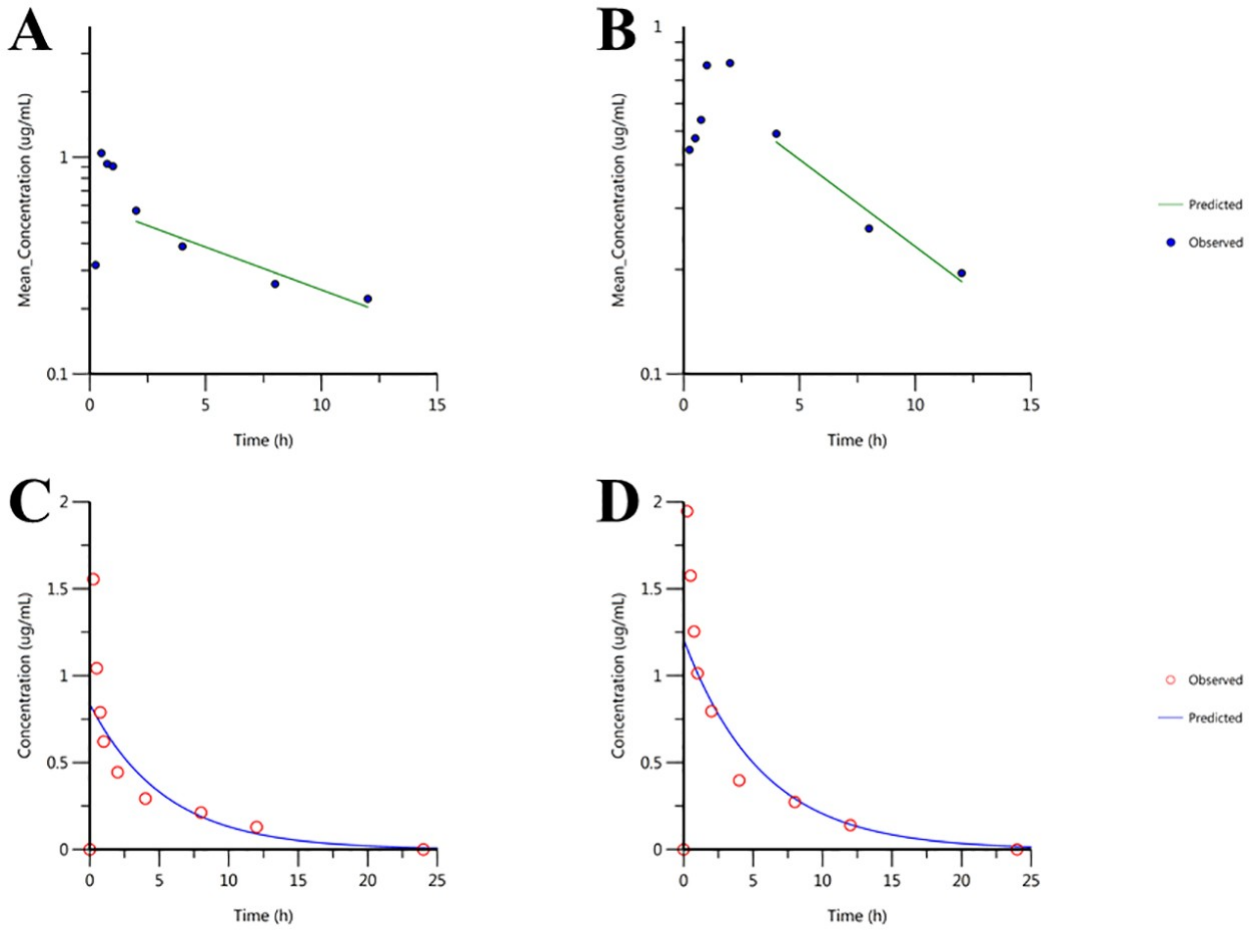
Pharmacokinetics plot of marbofloxacin alone and in combination with methyl gallate administered after PO and IV routes at different time. A) MAR after oral administration B) MAR in combination with MG after oral administration C) MAR after intravenous administration D) MAR in combination with MG after intravenous administration; *n = 10*.

The effect of MG on the in vivo PK of MAR was determined using healthy rat models after administering MAR alone and in combination with MG (Fig 2). The half-life of MAR administered orally alone and in combination with MG was 7.62 and 6.01 h, respectively. The time required to reach the highest concentration (T_max_) was 0.5 and 2 h, whereas, the maximum concentration (C_max_) was 1.04 and 0.78 μg/mL for rats treated with MAR alone and combined with MG, respectively. The overall AUC was 5.98 and 6.1 h.μg/mL for rats treated with MAR alone and in combination with MG, respectively (Table 2). A significant difference was observed for half-life (P = 0.0185), T_max_ (P<0.0001), and volume of distribution. However, no significant difference was observed between the two treatment groups for C_max_ and AUC (P>0.05).

**Table 2:** Pharmacokinetics parameters of marbofloxacin and methyl gallate after oral administration alone and in combination with methyl gallate in rats.

Parameter	Units	MAR	MGM
**HL_Lambda_z**	h	7.62±0.37	6.01±0.5*
**T_max_**	h	0.5±0.06	2±0.1*
**C_max_**	ug/mL	1.04±0.4	0.78±0.2
**AUCall**	h*ug/mL	5.98±1.6	6.11±1.3
**Vz_F_obs**	mL/kg	7759.03±145.1	6534.15±83.9*
**Cl_F_obs**	mL/h/kg	705.42±48.7	753.98±58
**MRT_last_**	h	4.51	4.46

HL_Lambda_Z: half-life; T_max_: time to reach the maximum concentration; C_max_: maximum concentration; AUC: Area under the curve; Vz: volume of distribution; CL: total clearance; MRT_last_: Mean residual time; MAR: marbofloxacin and MGM: Combination of marbofloxacin and methyl gallate. n = 10.

* indicates significance difference (P<0.05).

The half-life of MAR administered intravenously alone and in combination with MG was 3.77 and 3.93 h, respectively. The overall AUC was 4.54 and 6.86 h.μg/mL for rats treated with MAR alone and in combination with MG, respectively. The clearance rate was 1099.4 and 729.1 mL/h/kg whereas, the steady-state volume of distribution was 5985.6 and 4134.3 mL/kg, which is significantly different (P = 0.009) after MAR was administered alone and in combination with MG, respectively (Table 3). The bioavailability (F) of MAR in rats given MAR alone and in combination with MG was 131 and 89.1%, respectively.

**Table 3:** Pharmacokinetics parameters of marbofloxacin and methyl gallate after intravenous administration alone and in combination with methyl gallate in rats.

Parameter	Units	MAR	MGM
**V**	mL/kg	5986±620	4134.3±358*
**K10**	1/h	0.18±0.07	0.18±0.05
**AUC**	h*ug/mL	4.55±1.42	6.86±1.81
**K10_HL**	H	3.77±1.36	3.93±1.19
**C_max_**	ug/mL	0.84±0.09	1.21±0.1*
**CL**	mL/h/kg	1099.4±345	729.1±192
**MRT**	H	5.44±1.96	5.67±1.7
**Vss**	mL/kg	5985.98±620	4134.3±358

V: volume of distribution; K10: elimination rate; AUC: Area under the curve; K10_HL: half-life of the elimination phase; C_max_: maximum concentration; CL: total clearance; MRT_last_: Mean residual time; Vss: Volume of distribution at the steady state n = 10.

* indicates significance difference (P<0.05).

**Fig 3:**
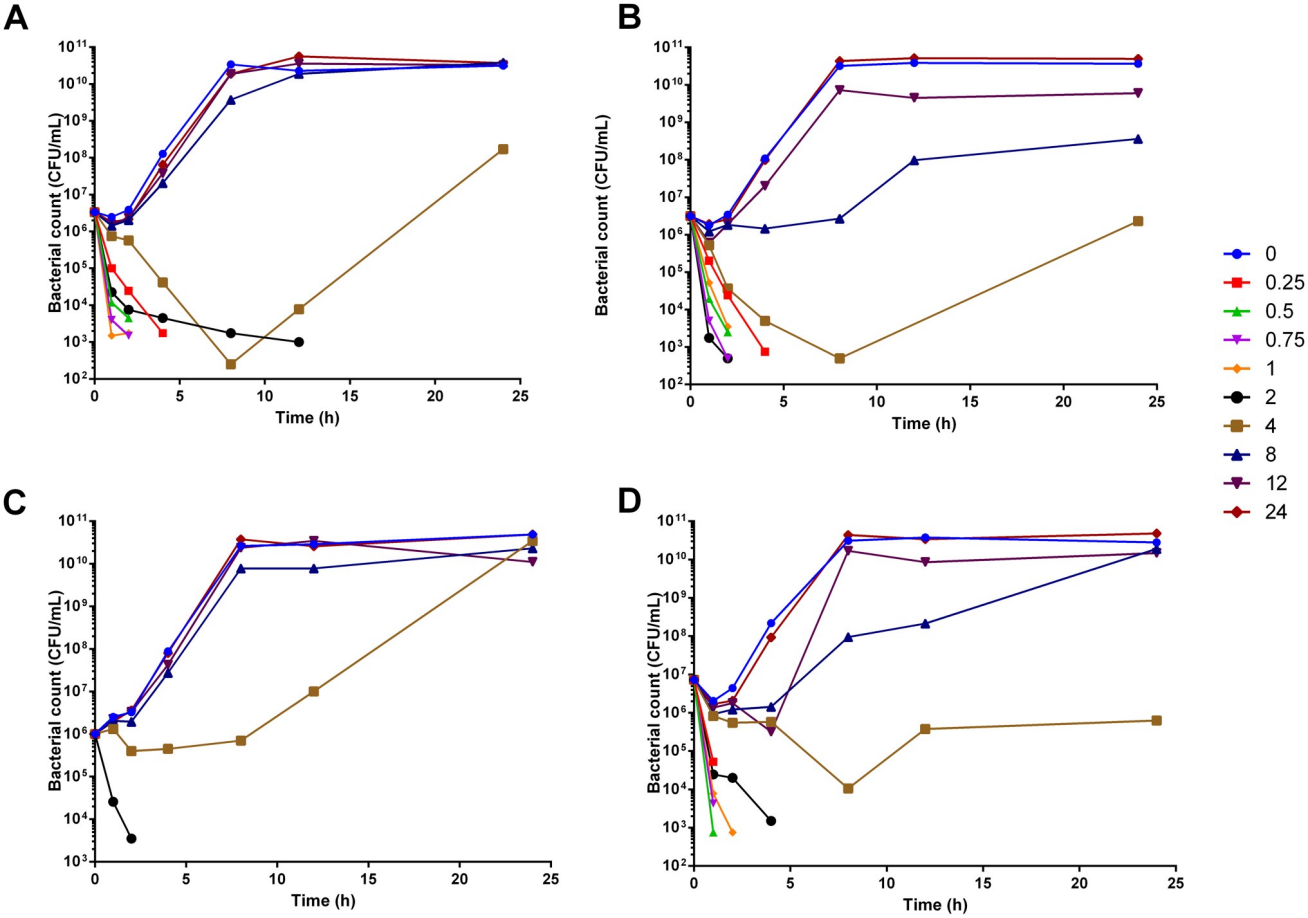
Time-kill assay of plasma after treatment with MAR and MG. A) MAR alone given orally; B) MAR combined with MG given orally; C) MAR alone given IV and D) a combination of MAR and MG given IV; *n = 6*.

**Fig 4:**
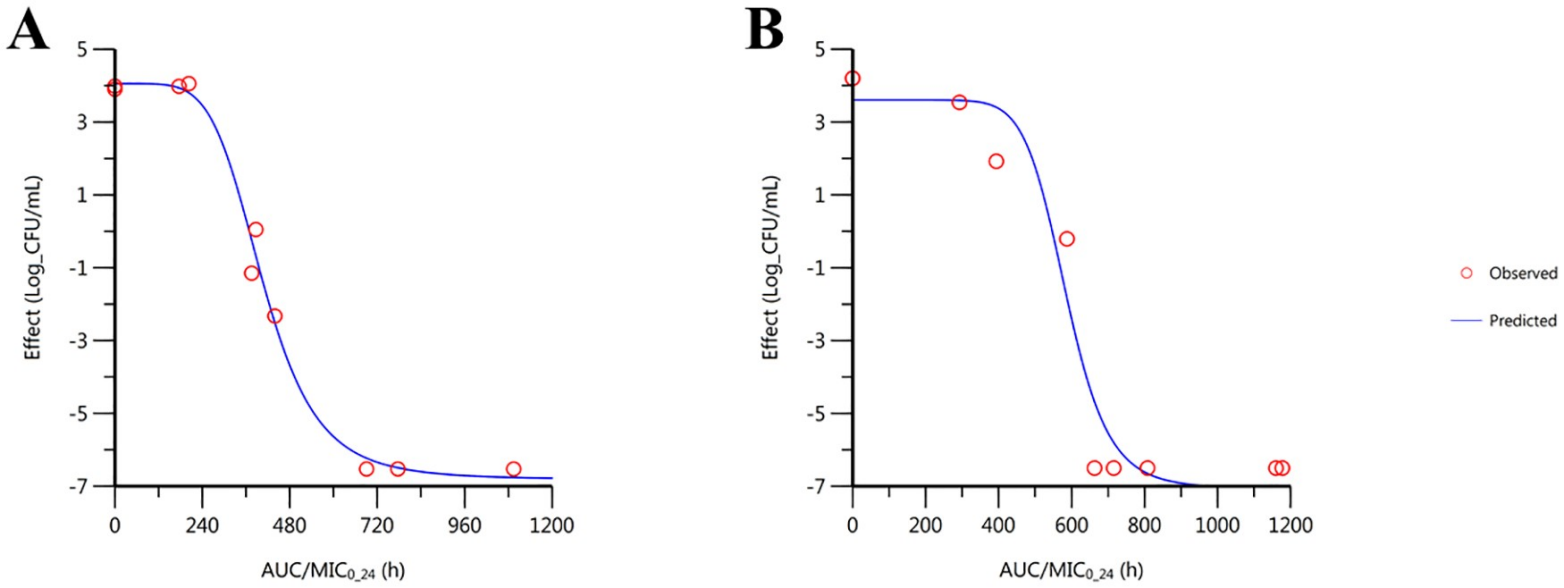
Ex-vivo sigmoidal inhibitory effect of MAR alone and in combination with MG. A) MAR administered orally alone B) MAR administered orally in combination with MG; n = 6.

In the *ex vivo* experiment, the bacterial inhibitory activity of plasma from rats orally given MAR alone observed from time-points 0.25 to 2 h (Fig 3A). Whereas, in combination with MG the bacterial inhibitory activity was extended from 0.25 h to 4 h. The bactericidal activity for orally treated rats was achieved within 4 h of incubation for the plasma collected from time-point between 0.5 to 2 h, (Fig 3B). The bacterial inhibitory effect was observed from time-point 0.25 to 2 h in rats treated with MAR alone and combined with MG after IV administration (Fig 3C and 3D). The mean slope of the curve of the AUC_0-24_/MIC is 5.4 and 10 for MAR given alone and in combination with MG. Whereas, the I_max_ for MAR alone and in combination with MG was 10.9 and 10.7, respectively. The IC_50_ was 406 and 504.8, respectively. The efficacy of MAR was increased when it is administered in combination with MG (Fig 4, Table 4).

**Table 4:** Antibacterial effect of marbofloxacin administered alone and in combination with methyl gallate after oral administration.

Parameter	MAR	MGM
**Imax**	10.86±0.58	10.66±1.14
**IC_50_**	405.99±11.78	584.75±30.06**
**E_0_**	4.05±0.29	3.6±0.67
**Gamma**	5.41±1.46	10±5.51

Imax: maximum inhibitory response (difference in the log of number of bacteria (CFU/mL) in the control sample); IC_50_: Concentration at which 50% of maximum inhibition; E_0_: the difference in the log number of bacteria in the sample treated with MAR between 0 and 24; Gamma: Exponent for sigmoid Imax model. MAR: marbofloxacin and MGM: Combination of marbofloxacin and methyl gallate. n = 6.

* indicates significance difference (P<0.05).

The integration of the PK/PD after oral administration showed that the AUC_24_/MIC of MAR after treatment with MAR alone and in combination with MG was 191.22 and 381.93, respectively. The C_max_/MIC ratio was 33.39 and 49.06 for MAR administered orally alone and in combination with MG after oral routes of administration, respectively (Table 5). A statistically significant difference was observed.

**Table 5:** Pharmacokinetics/pharmacodynamic indices of marbofloxacin and its dosage administered alone and in combination with methyl gallate after oral administration.

	MAR	MGM
**AUC_24_/MIC (h)**	191.22	381.93*
**C_max_/MIC**	33.39	49.06*
**Bacteriostatic dosage (mg/kg)**	6.20	3.70
**Bactericidal dosage (mg/kg)**	7.65	4.16
**Bacterial elimination dosage (mg/kg)**	8.3	4.34

AUC: Area under the curve; MIC: Minimum inhibitory concentration; C_max_: maximum concentration; MAR: marbofloxacin and MGM: Combination of marbofloxacin and methyl gallate.

* indicates significance difference (P<0.05).

The calculated dosage showed that the combination of MAR with MG reduces the given dosage by 40% in rats. The bacteriostatic dosage for MAR is 6.2 mg/kg, whereas in combination with MG it reduced to 3.7 mg/kg. Moreover, the bactericidal and bacterial elimination dosage decreased from 7.7 to 4.2 mg/kg and from 8.3 to 4.3 mg/kg for MAR administered alone and in combination with MG, respectively (Table 5).

## Discussion

Marbofloxacin has been used for the treatment of various infectious agents in veterinary medicine. In addition, several reports are available on their PK and PD indices in various animal species [12,30,31]. Recently, we have indicated that MG produced a synergistic effect in combination with MAR in preventing *Salmonella* Typhimurium invasion and intracellular survival [23]. However, no reports are available concerning the effect of MG on the PK of MAR. Hence, in this study, we investigated the antimicrobial activity of MAR and MG against the field and standard strains of *Salmonella* Typhimurium and the effect of MG on the PK of MAR using healthy rats. In addition, we determined the PK/PD integration of MAR and MG since this provides information about the optimal dosage, the antibiotic effect and reducing drug resistance [32].

A previous study showed that the maximum plasma concentration after intraperitoneal administration of 20 mg/kg of MG is 6.4 μg/mL [24]. However, this concentration is much smaller than the MIC of MG against *Salmonella* Typhimurium. Hence, we did not include the PK of MG in this study. However, if MG is administered orally, due to the limited absorption, it can only act locally in the small intestine where *Salmonella* Typhimurium resides. As we previously indicated, MG can inhibit *Salmonella* Typhimurium adhesion and invasion in the cells of the intestine and macrophages [23]. Hence, the antibacterial effect of MG will be more significant locally in the intestine than being systemic. However, this phenomenon should be supported by clinical trials in the future.

The antimicrobial activity of MG against various bacterial agents, including MRSA, *Escherichia coli*, *S*. enteritidis, *Vibrio cholerae*, *Shigella* species has been reported previously [17,18,20,21,33]. In this study, we have also confirmed its antimicrobial activity using a standard control and field strains of *Salmonella* Typhimurium isolated from clinical infections of pigs. In all the strains, MG has shown similar antimicrobial activity with the same concentration. The results are comparable with those observed in *E*. *coli* (250 μg/mL), and *Salmonella* strains (500 μg/mL) [17]. However, our MIC results are relatively higher than those reported in *Shigella* species (128–256 μg/mL) [20], MRSA (50 μg/mL) [21] and *Salmonella* strains from Chicken and human (3.9–125 μg/mL) [19] and also lower than those recorded in *Klebsiella oxytoca* (1000 μg/mL), *Actinomyces viscosus* (1000 μg/mL), *Streptococcus mutans*, *S*. *sobrinus* (2000–4000 μg/mL), and *Lactobacillus* species (8000 μg/mL) [16,17]. The observed differences might be due to variation in the bacterial strains, hosts, and epidemiology of the study area.

*Salmonella* Typhimurium resistance to fluoroquinolones is emerging and becomes a global concern [34]. Frequent usage and overdosage of a given antibiotic are related to the incidence of drug resistance. Hence, in addition to appropriate dosage formulation, drug combination is critical and effective in certain types of infections in reducing the risk of occurrence of multi-drug resistance strains [3]. In our study, it is shown that the presence of MG has increased the efficacy of MAR, and their combination showed a synergistic effect against both susceptible and relatively resistant field isolates of *Salmonella* Typhimurium. Our result agrees with previous reports which showed the synergistic effect of the combination of MG with nalidixic acid and ciprofloxacin [16,17]. This synergism has significant importance in reducing the antimicrobial resistance specifically for MAR or related antimicrobials [35]. MG has been shown to destabilize bacterial cell membrane integrity and affects the membrane potential, which results in an influx of antimicrobials, and might contribute to the synergism effect of both compounds [20,36,37].

Furthermore, the early killing of bacteria in the presence of MG contributes to reducing the contact time of the bacteria with MAR. The continuous exposure of microbial agents to concentration-dependent antibiotics increases the rate of development of resistance which can transfer to other antimicrobials either in the same or different groups [30]. Hence, reducing the exposure time of the bacteria with the antibiotic, as seen in MAR in combination with MG, could prevent the emergence of drug-resistant strains.

The AUC of MAR has increased upon the combination with MG whereas, the C_max_ was slightly decreased without statistical significance difference after oral administration. The T_max_ of MAR also increased when combined with MG. T_max_ shows the extent and rate of drug absorption and the higher T_max_ might result in the slow absorption of the drug. Even though the rate is different, the total amount of absorption, as seen in the AUC, which indexed the drug exposure of the body and its absorption did not show a significant difference [38]. This might be due to a slow rate of absorption due to increased T_max_ and lower volume but even distribution of MAR. In addition, the slight decrease in the rate of clearance, the increase in the half-life and mean residence time (MRT) of MAR indicates that MG might protect the degradation of MAR and can be used in improving the dosing regimen of MAR [39]. The short half-life observed for the combination of MAR and MG can have a near-instant effect and can be used for acute infections. Besides, it helps to reduce residual side effects which can be seen in drugs with long half-lives. This can further decrease the toxicity of MAR which can arise due to increased dosage. MAR has a higher percentage of bioavailability after oral administration [40]. This higher bioavailability is not significantly affected by the co-administration of MG.

After an intravenous administration, the volume of distribution and C_max_ showed a significant difference. The highest concentration achieved for IV administered drugs is equal if the equal dosage is given [41]. In this study, the same dosage regimen was given for both IV and orally administered drugs. Hence, there is no need to consider the C_max_ for the IV given drug. The volume of distribution of MAR increased when administered alone in comparison to the combined drugs after IV administration. Since dosing is proportional to the volume of distribution, it can indicate the extent of drug distribution and aid in the determination of dosage requirements. This could suggest that the MAR is distributed in the tissue and more dosage might be required to increase the plasma concentration when administered alone. In contrast, the decrease in the calculated dosage for the combination of MG and MAR might be attributed to the decrease in the volume of distribution. This also suggests that MG might reduce the dilution of MAR in the plasma or it decreased the plasma protein binding of MAR.

In combination with MG, the antibacterial activity of MAR in the plasma has increased significantly and was sufficient to kill the bacteria immediately after administration. This could attribute to the less protein binding ability of MAR. In which the presence of MG might further decrease its plasma protein binding activity and contribute to its increased antibacterial activity [12].

The *in vitro* PD results alone are not sufficient for clinical trials and applications. Thus, its integration with the *in vivo* PK properties of the drugs should also be evaluated [42]. In addition, the AUC/MIC surrogate marker is the best PK/PD index to describe the efficacy and give a better predictive value of MAR [11,12]. Hence, in this study, we determined the integration of PK/PD surrogate markers. The AUC/MIC ratio obtained in MAR administered in combination with MG was relatively higher than the MAR given alone. Even though, the ratios of both MAR alone and in combination with MG exceeded a value of 125, which is a recommended value to avoid the risk of resistance induction in fluoroquinolones, the ratio near to the value of 400 shows an optimal condition [12]. Furthermore, the C_max_/MIC ratio is 8–10 times higher than the conventional surrogate value for Gram-negative bacteria. Moreover, the obtained C_max_ showed a higher value than the MIC of each strain examined, showing the efficacy of the drug to kill the bacteria with sufficient concentration in the plasma.

The computed dosage showed that the presence of MG helps to reduce the total amount of MAR to be administered in comparison to administering MAR alone with better efficacy in rats. This could also apply in other species of animals. Even though decreasing the dose of antibiotics for economic benefits or misguided by PK/PD perceptions will decrease the AUC/MIC ratio and increase the pressure for the occurrence of drug resistance, the risk can be avoided by the combination therapy [11]. In general, our data suggested an increase in the potency and decrease the risk of development of antimicrobial resistance against MAR when combined with MG [12].

## Conclusions

In conclusion, in this study, we investigated the effect of MG on the PK of MAR, and its PK/PD integration with MAR for the first time using *in vitro*, *ex vivo* and *in vivo* rat experiments as a preclinical trial of the combination of MG and MAR. The *in vitro* experiments showed a synergistic activity of MAR and MG. Furthermore, the combination of MAR with MG increased its *ex vivo* antibacterial effects without affecting the PK of MAR. The detection limit of MG in the plasma after oral administration of a combination of MG and MAR at the 20 mg/kg dose was very low. This indicates that a relatively small amount of MG was absorbed. These could attribute to the insignificant effect of MG on the pharmacokinetics of MAR. This has a paramount significance in lowering drug resistance, which occurs by frequent use of a single antibiotic and its application in a higher dosage. In addition, a dose-use regimen for antimicrobial therapy in rats has recently been proposed. However, in the current study, we have used a low number of sample size and bacterial strains. Hence, we recommend further investigation of the PK and PK/PD integration of MAR and MG in clinically challenged animals and population PK studies in natural hosts. The low detection limit of MG in the serum samples was the major challenge we observed in this study. Hence, we recommend further study for the quantification of MG from serums. Besides, further study on the mechanism of action of the combination of the two compounds should be carried out.

## Supporting information

S1 Fig Chromatogram and standard curve plasma with MAR. A) Chromatogram of MAR after plasma was treated with 10 μg/mL of MAR B) The standard curve of MAR after two-fold dilution in free plasma.
(TIF)

## Abbreviations

ATCC: American Type Culture Collection
AUC: Area under the Curve
CFU: Colony Forming unit
CL: clearance
F: bioavailability
FIC: Fractional inhibitory concentration
HPLC: high-performance liquid chromatography
LOD: limit of detection
LOQ: limit of quantitation
MAR: Marbofloxacin
MBC: minimum bactericidal concentration
MG: methyl gallate
MIC: minimum inhibitory concentration
PD: pharmacodynamic
PK: pharmacokinetics
QC: quality control
Vss: Steady state volume of distribution
