# Supplementary figures and images for: Evaluation of the pharmacokinetic-pharmacodynamic integration of marbofloxacin in combination with methyl gallate against Salmonella Typhimurium in rats
Source: PLoS One. 2020 Jun 4;15(6):e0234211. doi: 10.1371/journal.pone.0234211 (PMC7272065; doi:10.1371/journal.pone.0234211)

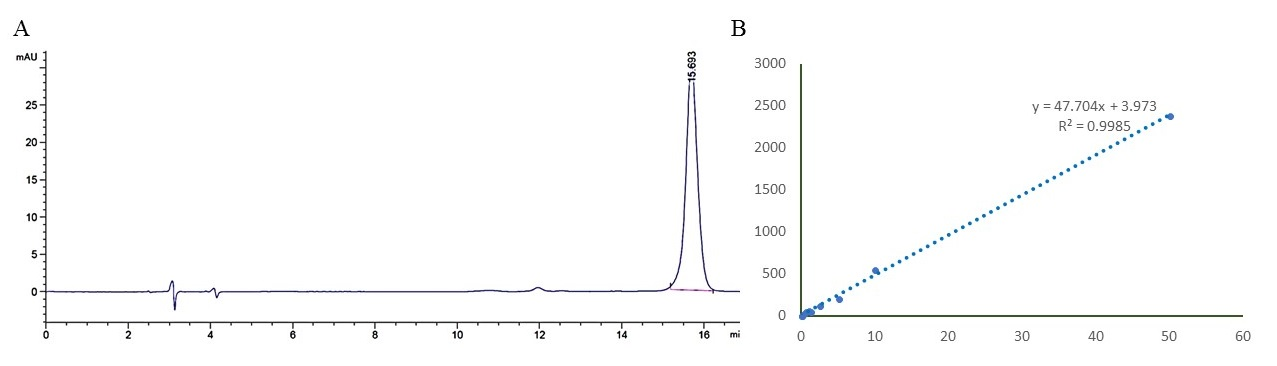

Supplement: S1 Fig — A) Chromatogram of MAR after plasma was treated with 10 μg/mL of MAR B) The standard curve of MAR after two-fold dilution in free plasma. (TIF) [file pone.0234211.s001.tif]
